# Supplementary material for: A high-resolution mRNA expression time course of embryonic development in zebrafish
Source: eLife. 2017 Nov 16;6:e30860. doi: 10.7554/eLife.30860 (PMC5690287; doi:10.7554/eLife.30860)
Supplement: Supplementary file 6. [file elife-30860-supp6.zip › biolayout-clusters-files/Cluster161-genes.html]

Cluster161


# Cluster161: Genes

| Ensembl ID | Gene Name | Chr | Start | End | Biotype |
| --- | --- | --- | --- | --- | --- |
| ENSDARG00000032963 | her12 | 23 | 21518371 | 21520434 | protein\_coding |
| ENSDARG00000054562 | her15.1 | 11 | 41370256 | 41371807 | protein\_coding |
| ENSDARG00000054560 | her15.2 | 11 | 41363460 | 41387324 | protein\_coding |
| ENSDARG00000030289 | jag1a | 1 | 50461185 | 50513861 | protein\_coding |
| ENSDARG00000056130 | neurog1 | 14 | 26405226 | 26406724 | protein\_coding |
| ENSDARG00000017107 | nr2e1 | 20 | 32534517 | 32543527 | protein\_coding |
| ENSDARG00000007430 | prdm12b | 5 | 62817114 | 62821531 | protein\_coding |
| ENSDARG00000103442 | si:ch211-193l2.3 | 11 | 41430444 | 41432228 | protein\_coding |
| ENSDARG00000103339 | si:ch211-193l2.5 | 11 | 41423596 | 41424874 | protein\_coding |
